# A quasi-randomised pilot study on the efficacy and perceived usefulness of adding chemosignals to mindfulness practice for women with social anxiety

**DOI:** 10.1038/s41598-025-18246-w

**Published:** 2025-09-12

**Authors:** Emma T. Eliasson, Elisa Vigna, Elisa Dal Bò, Cinzia Cecchetto, Letizia Zurlo, Claudio Gentili, Enzo Pasquale Scilingo, Alberto Greco, Fabio Di Francesco, Luca Citi, Nicola Vanello, Johan N. Lundström, Gergö Hadlaczky, Vladimir Carli

**Affiliations:** 1 National Centre for Suicide Research and Prevention of Mental Ill-Health (NASP), Karolinska Institutet, Granits väg 4, Stockholm, 17165 Sweden; 2 Department of General Psychology, University of Padua, Via Venezia 8 - 35131, Padua, Italy; 3 Padova Neuroscience Center (PNC), University of Padua, Via Orus 2/B - 35131, Padua, Italy; 4 Department of Information Engineering, University of Pisa, Pisa, 56126 Italy; 5 Research Center “E. Piaggio”, University of Pisa, Largo Lucio Lazzarino 1, Pisa, 56122 Italy; 6 Department of Chemistry and Industrial Chemistry, University of Pisa, Pisa, 56124 Italy; 7 School of Computer Science and Electronic Engineering, University of Essex, Colchester, UK; 8 Department of Clinical Neuroscience, Karolinska Institutet, Nobels väg 9, Stockholm, 17177 Sweden

**Keywords:** Chemosignals, Social anxiety disorder, Mindfulness, Olfaction, Chemical biology, Psychology

## Abstract

Body odours (BOs) of individuals in specific emotional states can influence receivers’ responses – referred to as an emotional contagion. To investigate the potential of BOs to enhance the effects of mindfulness practice, this quasi-randomised pilot study tested the hypothesis that participants exposed to emotional BOs during mindfulness meditation would exhibit a steeper decrease in state anxiety symptoms compared to mindfulness alone (clean air control). Ninety-eight women meeting criteria for Social Anxiety Disorder (SAD) received two mindfulness sessions over two consecutive days, while randomly allocated to one of four conditions: fear BO, joy BO, neutral BO or a clean air control group. No odour × time interaction effect was observed, rejecting the primary hypothesis. Although not statistically significant, effect size estimates suggested a greater reduction in state anxiety for the group receiving fear chemosignals (Day 1 Cohen’s *d* = 0.26, Day 2 Cohen’s *d* = 0.54) compared to the clean air control group. Moreover, the BO groups perceived the mindfulness practice as significantly more helpful compared to the control group (*p* = 0.002). Given the sample size limits, a larger Randomized Controlled Trial (RCT) incorporating more mindfulness + BO sessions is recommended to further examine the therapeutic potential of human BOs.

**Supplementary Information:**

The online version contains supplementary material available at 10.1038/s41598-025-18246-w.

## Introduction

Chemical signals in human body odours (BOs) hold the potential to convey an array of biological and social information to our surroundings^1–4^. BOs have been shown to be modified by changes in physical activities and blood vessel constriction which the autonomic nervous system regulates unconsciously under various emotional or psychological conditions^5,6^. Chemosignals of individuals, or ‘senders’, in a particular emotional state have been shown to produce congruent emotional responses in ‘receivers’, a phenomenon that has been described as an emotion contagion^5,7,8^. This occurrence was first demonstrated for negative emotions including disgust, aggression, and fear^7^ but has subsequently also been shown for positive emotional states^9,10^. Responses to BOs have been measured through a variety of means, including changes in facial muscle activity^11–13^, cognitive task performance^14^, brain activity^1,15^, heart rate variability^10,16,17^, creativity^10^ and self-reported affect^18^. However, whilst a few studies have included individuals with psychiatric disorders^17,19–23^, there is a paucity of research investigating if BOs can be used to help regulate emotional states or increase well-being; findings which could have implications for psychiatric treatments^6,24^. This may be particularly relevant for conditions that poses challenges to social interactions and navigation of social information from others, such as social anxiety^25^.

Social Anxiety Disorder (SAD) is characterized by feelings of fear and anxiety during social interactions, accompanied by an intense worry of being negatively evaluated by others^26^. SAD can be a detrimental condition, with the negative impacts penetrating many life aspects including work, social and private life^27^. Along with depression and other anxiety conditions, SAD is among the most prevalent mental disorders^28^, with survey data indicating increasing rates among young adults^29^. Cognitive Behavioural Therapy (CBT) is deemed the most effective and first-line therapy for SAD^30–32^. However, due to its resource intensity, access to CBT remains limited^33^, highlighting a need to provide effective yet accessible treatment options. Moreover, treatment attrition is high for this patient group^34^ whilst recovery rates are modest^35^. One category of therapies that have gained increased popularity are mindfulness-based treatments^30^. However, whilst mindfulness treatments have been deemed effective for individuals with social anxiety disorder, their effectiveness in contrast to CBT remain modest^30^, even though it should be noted that the differentiation between mindfulness based therapies and CBT can be ambiguous as mindfulness techniques are often integrated within CBT programmes to enhance outcomes^36,37^. Nevertheless, mindfulness can be advantageous due to its wide accessibility and ease of administration, with the potential to be delivered through guided self-help^38^. This is supported by findings indicating that mindfulness-based treatments delivered online are associated with reduced anxiety symptoms, even though effects are in the small range^39^. Therefore, finding ways of enhancing the benefits of mindfulness practice regarding anxiety symptom alleviation would be beneficial, particularly in light of their wider accessibility, in comparison to more traditional therapeutic approaches.

Given this need, our research group recently conducted an exploratory study, investigating the prospective advantage of adding BOs collected from individuals in fearful or happy emotional states, to two brief mindfulness sessions^24^. Findings indicated that both fear and happiness BOs enhanced state anxiety-reducing effects of the mindfulness practice for individuals meeting criteria for SAD (*n* = 48). The apparent benefits of both fear and happiness BOs, albeit somewhat contradictive of the emotion contagion hypothesis^7,12,13,18,40^, led to the interpretation that BOs, irrespective of the emotional condition in which they were collected, may carry the potential to reduce anxiety symptoms through conveying a human presence^24^. This interpretation aligns with research indicating that whilst social interactions are often anxiety-inducing, individuals with SAD appear to show positive affect in the presence of others in contrast to spending time alone^41^. Likewise, elevated anxiety symptoms have been associated with an increased desire to approach others^42^.

In light of our promising findings^24^ and the importance of improving alternative treatments for SAD, we report here a follow-up, hypothesis-driven pilot study to test whether BOs extracted from individuals experiencing joyful, fearful, or neutral emotional states could be used to enhance the benefits of two mindfulness sessions in subjects meeting criteria for SAD, further investigating the role of chemosignals in mindfulness practice for this condition. Whilst previous studies^9,10^ including our pilot work^24^, used the term “happiness BO”, we reasoned the condition may more appropriately be described as “joy BO”, given the complexity of happiness as an emotional state^65^ and the participants’ ratings following the emotion induction, which rated this positive emotion using the terms joy and amusement (Table S1 in the supplementary material). Therefore, whilst we utilised similar emotion induction procedures as previous olfaction studies^9,10,24^ we termed this odour condition ”joy BO” in the present study. The neutral BO condition was included to build on our previous findings^24^ and further disentangle whether the emotional context in which the BOs were collected play a role. Our aim was to test the primary hypothesis that participants exposed to BOs (neutral, fear, and joy) in combination with two brief mindfulness sessions would exhibit a steeper decrease in anxiety symptoms compared to a control group undergoing mindfulness without exposure to BOs (hereinafter referred to as the clean air condition).

As secondary objectives, we aimed to explore the ability of BOs to strengthen resilience in response to a stressful event by introducing a stress induction after the two consecutive mindfulness sessions, to examine potential benefits of BOs at a follow-up assessment occurring one day after the mindfulness sessions, as well as evaluating over-all perceived benefit of the intervention among participants. Finally, given the pilot nature of the study, we aimed to use this study to guide the planning of a larger scale randomised controlled trial (RCT).

## Methods

This study was carried out within the EU Horizon2020 Project POTION (‘Promoting Social Interaction through Emotional Body Odours’) and conducted at the University of Padua, Italy.

### Ethical and safety considerations

The study was conducted in accordance with the declaration of Helsinki, and was approved by the local Ethics Committee, University of Padua (protocol no. 3113). Informed consent was obtained from all participants taking part in the study. A compensation of €13 was provided to participants for their involvement. All study assessments were conducted by trained research staff, and in the case of participants reporting significant mental health symptoms, contact details to relevant help services were provided.

### Design

The study utilized a between-subjects design, where participants with SAD underwent two 24-minute mindfulness meditation sessions while allocated to one of four odour conditions: joy BO, fear BO, neutral BO, or clean air. A single-blind procedure was employed, where study participants were blinded to the treatment allocation.

### Power-calculation and sample size

An a priori power analysis was conducted based on effect size extrapolated from the previous exploratory study conducted within the POTION project^24^. The power calculation was performed using G*Power software (version 3.1.9.7) with the function ANOVA, repeated measurements, between factors. The parameters entered were α = 0.05, 1 – β = 0.95, and effect size of between subjects’ analysis of the first trial day of the above-mentioned study (Cohen’s *f* = 0.3745), for a study design of 4 (odour condition; clean air, neutral BO, fear BO, joy BO) × 2 (time; pre, post). Based on these parameters, a total sample size of 96 was needed.

### Body odour collection and Preparation

Sweat samples were collected from a different group of participants, so-called BO donors, at the Instituto Superior de Psicologia Aplicada (ISPA, Lisbon, Portugal). The protocol for sweat collection was approved by the Ethics Committee of ISPA in September 2018. The sweat collection and stimulus composition procedures were similar to those used in our earlier study^24^, even though additional samples from donors having undergone non-emotional (neutral) emotion inductions were included. Sweat pads used for the current study were based on samples from 26 donors (14 males, mean age = 21.3, SD = 2.5 and 12 females, mean age = 21.6, SD = 3.6). A within -subjects design was used where each BO donor attended the lab at three separate occasions. These sessions were spaced out between days, with the median interval between sessions being 7 days (mean = 8.9), with a minimum of 4 days and a maximum of 27 days between collections. During each session a single emotional state (fear, joy or neutral) was induced, with the order of emotional conditions randomized across sessions. To induce relevant emotional states, donors watched 25-minute video clips that were fear inducing, joy inducing or were non-emotional nature documentaries, whilst sweat was collected via pads placed in the armpits. To ensure successful emotion induction, participants’ emotional states were assessed before and after (for results see supplementary material Table S1). As described above, whilst we decided to use the term “joy BO” instead of “happiness BO”, the change in terminology did not reflect a change in stimulus material, but rather a conceptual re-evaluation of appropriate terminology to describe the condition. Thus the joyful BO stimulus used for the current study was identical to the happiness BO stimulus described in our previous study^24^.To avoid sweat contamination, participants were asked to undergo a strict behavioural and dietary regimen two days before the body odour collection^7^. After the emotional induction, pads were removed, frozen at − 80 °C, shipped to the University of Padua (Italy) in dry ice and subsequently stored at − 80 °C. To circumvent potential sex-related, or other influences of individual donors on odour perception^43^, a super-donor design was used during preparation of the samples for the present study. Each super donor sample included body odour from four different individuals (two males and two females randomly selected, with one male and one female contributing odour samples from the left armpit, and the other male and female from the right armpit), with only one-eighth of collected pad per individual used in the final super donor pool. Similar procedures for collection, storage and preparation have been used and validated in previous studies^40,44^.

### Recruitment and enrolment criteria

Recruitment to the study commenced on the 17/10/2022 and remained open until the recruitment target was set, with the final participant completing the follow-up assessment on the 20/05/2023. Researchers at the University of Padua disseminated the study information via flyers, and individuals expressing an interest were invited to undergo an initial online screening process on the Qualtrics platform. The following inclusion criteria were assessed: (1) female sex, (2) aged 18–35, (3) non-smoker, (4) scoring ≥ 30 on the Liebowitz Social Anxiety Scale in its self-report formulation (LSAS-SR^45^. The decision to include only women was taken to avoid sex-related confounds regarding olfactory abilities, where women generally outperform men^46,47^. Participants were ineligible if they: (1) had chronic rhinitis or any other condition potentially impacting odour perception; (2) were pregnant or breastfeeding; (3) reported a diagnosis of mental disorders (including substance abuse disorders) other than social anxiety disorder; (4) reported having severe somatic or neurological conditions; (5) reported current use of psychotropic medications (including antidepressants, antipsychotics, anxiolytics, and mood stabilizers); (6) were presently undergoing psychological therapy; (7) had severe psychotic symptoms (i.e. hallucinations and/or delusions); (8) reported presence of suicidal thoughts or; (9) were unable to provide informed consent.

Participants meeting initial screening criteria were extended invitations for a clinical interview. Within this interview, the presence of social anxiety symptoms was validated using Module F of the Structured Clinical Interview for DSM-5 (SCID-5-CV^48^, . Furthermore, anosmia or hyposmia were tested using the Sniffin’ Sticks test^49^ to ensure intact olfactory abilities (for a description see Supplementary Materials). The final study sample included 98 women (fear BO *n* = 24, joy BO *n* = 25, neutral BO *n* = 25, clean air *n* = 24).

### Quasi-randomisation procedure

Allocation of the odour conditions was conducted by research investigators at the site where the study was undertaken (University of Padua), and was done using Excel, creating a list of randomised odour conditions to be used for the study. Allocation to treatment groups occurred in a quasi-randomised manner, as allocation to the odour conditions depended on the day of participation, where those participating on the same day were allocated to the same odour condition (with a maximum of two participants being tested in one day). This procedure was chosen for the pragmatic reason that we aimed to enable optimal use of the sweat pads, given these needed to be discarded after one day of use.

### Study procedure and outcome measures

**Fig. 1:**
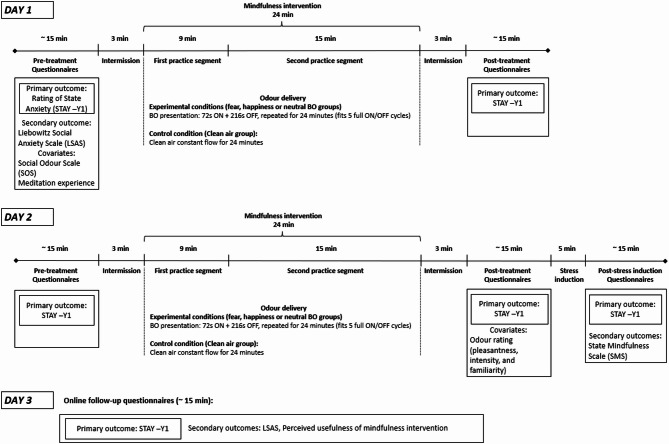
Schematic outline of the study procedure.

Following informed consent and screening, participants were assigned to one of the four odour conditions in which they remained throughout all experimental sessions. The intervention was performed over two consecutive days, with the experimental procedure, including data collection and mindfulness practice, lasting approximately 60 min per day. One hour prior to each session, participants were instructed to abstain from consuming food and beverages, excluding water. Figure 1 outlines the study procedure and questionnaires collected at each time-point.

#### Study intervention and stress-induction

The intervention took place at the University of Padua. The mindfulness training was done individually and performed using two mindfulness practices presented through recorded audio tracks available from a smartphone app called “Con tatto” (developer LifeSTech research team). The practices focussed on conscious breathing, meditation and relaxation exercises. The first practice segment (“The breath that frees”) was 9 min long whereas the second practice (“The thin breath”) was 15 min long. Throughout the exercises participants were asked to be present in the moment, and to observe their bodily sensations and their breathing. All participants listened to the guided mindfulness practice via noise cancelling headphones. The total length of the practice was 24 min. Throughout the mindfulness practice, BOs or clean air were delivered with a custom-built^50^ continuous airflow, computer-controlled olfactometer, directly to both nostrils with a nasal cannula during the intervention. Airflow was kept constant between 50 and 70 ml/min, BOs were delivered in 72 s-long pulses separated by a 216 s clean air. As indicated in Fig. 1, following the mindfulness session on day 2, participants underwent a stress-induction where they were given an abstract, and told they needed to give a presentation about the abstract to a small group of people, which they were given 3 min to prepare for. State anxiety levels were assessed before and after the stress-induction. At the end of the experimental session on day 2, participants were told that the presentation had been cancelled, and they did not need to present.

#### Primary outcome

*State-Trait Anxiety Inventory (STAI)*^51^: A 40-item self-report scale that assesses two forms of anxiety, state anxiety (STAI-Y1) and trait anxiety (STAI-Y2), 20 items each, on a four-point Likert scale (ranging from 1 (“not at all” in the State subscale STAI-Y1 and “almost never” in the Trait subscale STAI-Y2) to 4 (“very much” in the State subscale STAI-Y1 and “almost always” in the Trait subscale STAI-Y2), where higher scores indicate elevated levels of anxiety. For the current study, the 20-item STAY-Y1 scale was used, as we were interested in ratings of state anxiety levels. Scores range from 20 to 80, cutoffs are typically 20–39 for no or low anxiety, 40–59 for moderate anxiety and 60–80 for high anxiety^51^.

#### Secondary outcomes

*Perceived usefulness*: This was rated with one item asking participants how useful they thought the mindfulness sessions were and rated from 0 (“not useful at all”) to 10 (“very useful”).

*State Mindfulness Scale (SMS)*^52^: A 21-item self-report questionnaire assessing two state mindfulness aspects: one measuring state mindfulness of bodily sensations and the other reflecting state mindfulness of the mind, using a 5-point Likert scale from 1 (not at all) to 5 (very well), where a high score reflects a high state of mindfulness.

*The Liebowitz Social Anxiety Scale (LSAS-SR)*^45^: A 24-item questionnaire where fear and avoidance in a range of social and performance situations are assessed. It is rated on a 4-point scale, where a higher score reflects higher anxiety.

#### Covariates

*Social Odor Scale (SOS)*^53^: A 12-item self-report questionnaire designed to measure body odour awareness, including individual body odour, familiar and unfamiliar body odours, romantic partner body odour, and body odour of strangers. Items are rated on a 5-point scale, where a high score indicates higher body odour awareness.

*Odour ratings*: Subjects were asked to rate the intensity, pleasantness, and familiarity of the odour presented during the mindfulness intervention, each on a 10-point scale, where a higher score indicated higher odour intensity, pleasantness, and familiarity. Similar rating procedures have been used in previous olfaction studies (e.g^54^. To avoid drawing participants attention towards specific aspects of the olfactory content of the experiment as it was ongoing, questions regarding odour ratings were given after the experimental sessions were completed on day 2.

*Prior meditation experience*: To control for prior meditation experience the question “Do you practice, or have you ever practiced mindfulness meditation?” was asked to everyone. If yes, there was a follow-up question “how often do you practice?” with possible answers “not currently”, “practice less than one time a week”, “less than three times a week”, “practice three times a week or more”, “practice every day”.

### Trial registration

With recruitment commencing on the 17/10/2022, the trial was retrospectively registered on date: 16/11/2022 (ISRCTN Registry No. 98675422). The registration also included investigating physiological responses to BOs as secondary outcomes, results which will be reported in a separate publication. Due to unforeseen recruitment challenges, mainly brought on by the COVID-19 pandemic, screening criteria was made less stringent after study commencement, to increase the potential recruitment pool. This change was implemented on 19/12/2022 and entailed alterations to the LSAS screening threshold from ≥ 50 to ≥ 30. Whilst a score of 50 may have lessened the risk of floor effects, this reduction was deemed necessary to ensure timely recruitment. Importantly, the presence of SAD was still validated using Module F of the Structured Clinical Interview for DSM-5 (SCID-5-CV^48^. Similar LSAS thresholds have been set in other studies alleviating symptoms of social anxiety (e.g^55^).

### Statistical analysis

Data were analysed using R statistical software (version 4.1). Differences in demographic data, baseline characteristics and odour ratings were explored by means of one-way ANOVA or Kruskal-Wallis test when normality was violated. For descriptive purposes, the magnitude of change (mean difference and Cohen’s *d*) of pre- to post-treatment changes within groups, as well as between-groups comparisons, were assessed. These analyses were conducted separately for Day 1 and Day 2, as well as for the change from pre-treatment on Day 1 to follow-up on Day 3. Effect sizes were interpreted using conventional thresholds: d < 0.2 as negligible, 0.2 ≤ d < 0.5 as small, 0.5 ≤ d < 0.8 as medium, and d ≥ 0.8 as large^56^. Pairwise *t*-tests with Holm correction for multiple comparisons were employed to evaluate these changes. In addition, whilst the sample met diagnostic thresholds for social anxiety, the majority of participants were in the low to moderate range of state anxiety (STAI-Y1) at baseline. Given this baseline profile, we chose not to rely on severity categories for interpreting treatment effects. Instead, Jacobson & Truax^57^ method was used to assess whether STAI-Y1-score changes reached clinically meaningful levels.

#### Test of primary hypothesis

To examine the primary hypothesis, changes in state anxiety (STAI-Y1) scores were calculated through linear mixed modelling, where a 4 (Odour condition) × 2 (Day) × 2 (Time) model was tested. Odour condition (clean air vs. fear BO vs. joy BO vs. neutral BO) served as the between-subject factor, while time (pre-treatment vs. post-treatment) and day (day 1 and 2) were within-subject factors. Statistical models were applied through the *lme4* and *lmerTest* R packages. To control for variations in pre-treatment state anxiety scores, all models were fitted using random intercepts.

#### Secondary outcomes

Secondary outcomes (change following stress-induction, change at follow-up, perceived usefulness and state mindfulness scores) were explored by means of one-way ANOVA or Kruskal-Wallis test when normality was violated. Changes in STAI-Y1 scores after the stress induction and changes from pre-treatment on day 1 to the final follow-up assessment point on day 3 were examined through linear mixed modelling.

#### Covariates

Analyses were conducted with and without covariates. Because there were fewer data points for the SOS scale due to this scale being introduced at a later stage during the trial (data collected from 70 participants; clean air *n* = 16, fear *n* = 14, joy *n* = 17 neutral *n* = 23), three separate models were tested; one without the planned covariates, that included the full sample (Model 1), one where odour ratings and prior meditation experience were added as covariates (Model 2) and one where odour ratings and prior meditation experience and the SOS were included as covariates (Model 3). The outputs of all models are presented in the supplementary materials, and unless the addition of covariates substantially influenced the main results, the output of the analyses without the covariates are reported in the main text, as these analyses comprised the full sample.

## Results

### Sample characteristics

**Fig. 2:**
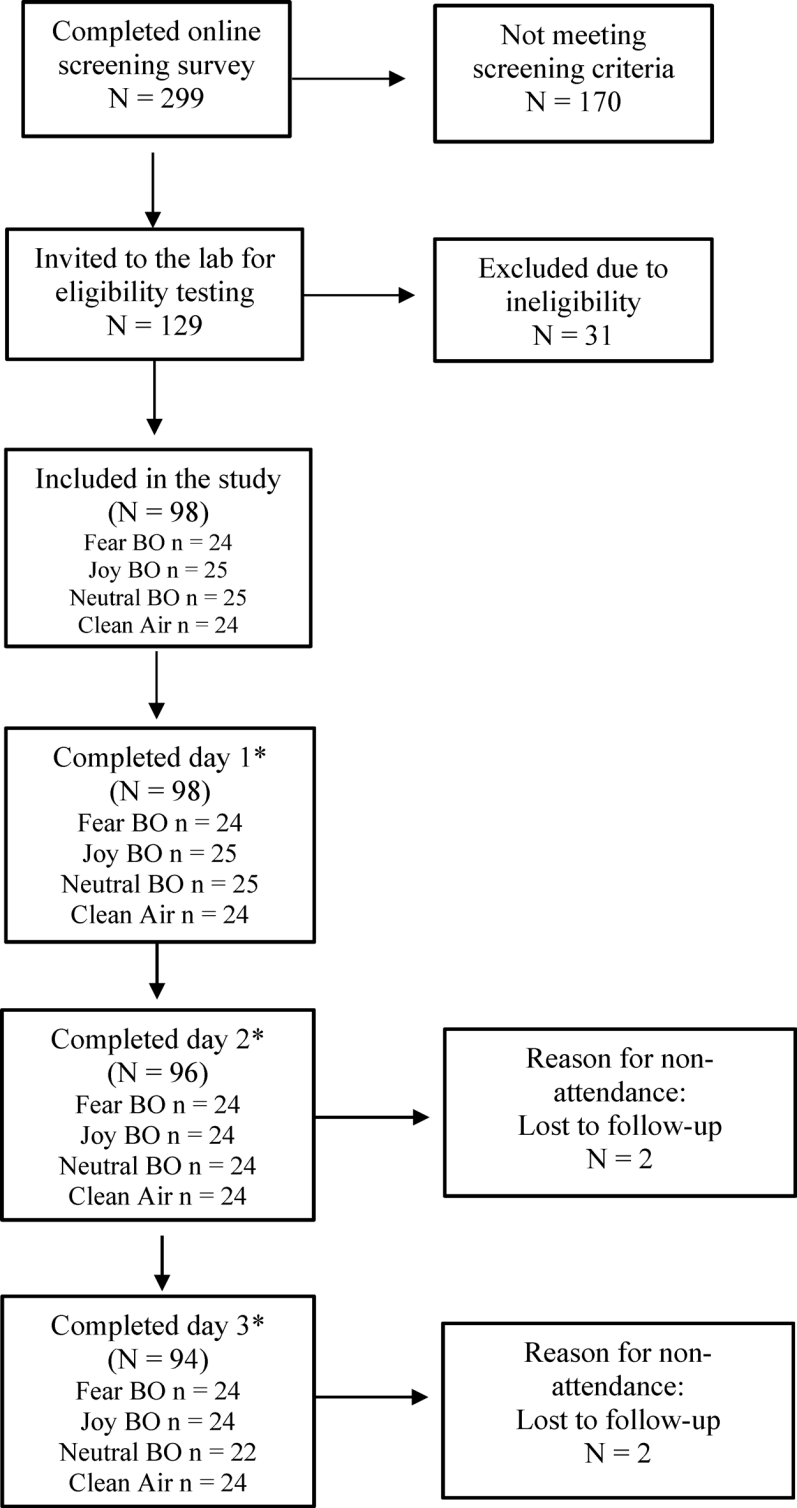
Consort diagram outlining participant recruitment and retention. *Refers to participants who completed the intervention and the primary outcome, or only the primary outcome on day 3 (follow-up).

Table 1 outlines demographic and baseline characteristics. One-way ANOVA comparing scores between each odour group indicated no significant differences in LSAS on day 1 (pre-treatment) and SOS scores, whereas marginally significant differences in age were recorded, with Holm corrected post-hoc tests indicating a difference between the fear and clean air condition (*p* = 0.02). Wilcoxon rank-sum tests (for two independent groups) were used to assess differences in odour ratings between the odour conditions pairs. The results showed no significant differences between most group pairs (all adjusted *p*-values = 1.0), with the sole exception of neutral BO vs. joy BO within the pleasantness condition (*p* = 0.73). These findings align closely with the Kruskal-Wallis test (Table 1), which did not indicate any global effect across groups. Regarding experiences of meditation, 73.2% (*n* = 71) reported having no prior experience, whereas 21.6% (*n* = 21), had practiced at least once in their life and 5.2% (*n* = 5) were regularly practicing. The consort diagram in Fig. 2 outlines the recruitment and enrolment process. Whereas a total of four participants (4.1%) were reported as lost to follow-up (two participants only attended the first intervention day, and another two participants did not complete the follow-up assessment on day 3), no participant actively withdrew from the study.

**Table 1:** Baseline characteristics and odour ratings (Mean and SD) of the four odour conditions.

	Fear *n* = 24	Joy *n* = 25	Neutral *n* = 25	Clean Air *n* = 24	*p*
Age	23.7 (2.6)	23.0 (1.4)	22.8 (1.7)	22.0 (1.7)	0.05^a^
Education^b^	16.0 (1.4)	16.1 (1.7)	16.3 (1.8)	16.0 (1.8)	0.90
LSAS	63.2 (18.7)	60.3 (18.6)	64.2 (19.0)	65.5 (19.3)	0.80
SOS^c^	27.1 (7.0)	26.5 (5.5)	30.8 (5.5)	26.5 (7.7)	0.09
STAI-Y1	42.3 (10.5)	36.9 (8.6)	37.4 (8.0)	42.4 (11.8)	0.14^a^
STAI-Y1 severity category, n (%)	L: 10 (45%) M: 9 (41%) H: 1 (5%)	L: 15 (63%) M: 7(29%) H: 1(4%)	L: 16 (64%) M: 9 (36%) H: 0 (0%)	L: 13 (54%) M: 8 (33%) H: 3 (13%)	
Familiarity	4.0 (2.8)	3.2 (2.7)	3.5 (2.7)	3.46 (2.6)	0.71^a^
Pleasantness	4.2 (2.8)	4.9 (2.8)	3.8 (2.5)	4.33 (2.9)	0.55^a^
Intensity	2.7 (1.5)	3.1 (1.8)	2.9 (2.1)	2.54 (2.1)	0.54^a^

^a^Calculated with Kruskal-Wallis test due to non-normally distributed data. Normally distributed data calculated using ANOVA. ^b^Reported in years. ^c^Data only collected from 70 participants (clean air *n* = 16, fear *n* = 14, joy *n* = 17 neutral *n* = 23), due to this scale being introduced at a later stage in the trial. ^d^Severity category: L = Low (20–39), M = Moderate (40–59), H = High (60–80).

### Magnitude of state anxiety symptom change

#### Within-group change

Mean difference scores of state anxiety (STAI-Y1) for each group as well as the magnitude of change for both treatment days, are presented in Table 2. Changes in state anxiety scores were tested within each group using paired-sample *t*-test with each day’s post-treatment scores compared to its respective pre-treatment scores. As depicted, the magnitude of change was large in the odour conditions for Day 1 in comparison to a medium effect observed for the clean air condition. Only the fear condition showed a large effect size change between pre-post treatment across both treatment days; somewhat also reflected in a larger proportion of participants achieving a clinically meaningful improvement in this group on both treatment days. Regarding change from pre-treatment on day 1 to follow-up on day 3, a small decrease in symptoms from Day 1 to follow-up (Day 3) was evident only in the fear group (*p* = 0.03).

#### Between-groups comparisons

The between-groups comparisons of changes in STAI-Y1 scores from pre- to post-treatment for day 1 and day 2 as well as from pre-treatment on day 1 to follow-up, using the clean air group as the reference condition, are shown in Table 3. Mean differences were calculated with a linear model using odour condition as fixed effect, with confidence intervals derived from the model coefficients. Assumptions of homogeneity of variance were assessed using Levene’s test, which indicated no significant variance differences between conditions. The fear condition appeared to have greater reductions in STAI-Y1 scores compared to clean air, particularly on day 2 and from pre-treatment on day 1 to follow up (day 3), reaching a medium effect size; even though this difference was not statistically significant (day 2: *p* = 0.07, day 3: *p* = 0.09). For the joy and neutral conditions, differences compared to clean air were negligible or small (*p*s > 0.10).

**Table 2:** Within-group change in STAI-Y1 scores for each odour condition for day 1, day 2 and at follow-up.

Condition	Mean diff. (95% CI)	*P*-value	Cohen’s d (95% CI)	Mag.	RC
Day 1 (pre-post)					
Clean Air	–6.3 (–9.7, − 2.8)	< 0.001	0.77 (0.41, 1.24)	M	33%
Fear	−8.2 (–11.3, − 5.1)	< 0.001	1.18 (0.88, 1.82)	L	45%
Joy	–5.5 (–8.2, − 2.9)	< 0.001	0.90 (0.49, 1.61)	L	21%
Neural	–6.7 (–9.5, − 3.8)	< 0.001	0.96 (0.55, 1.80)	L	28%
Day 2 (pre-post)					
Clean Air	–2.5 (–5.1, − 0.02)	0.048	0.43 (0.02, 1.02)	S	21%
Fear	–6.2 (–9.4, − 2.9)	< 0.001	0.84 (0.45, 1.46)	L	39%
Joy	–3.5 (–5.8, − 1.2)	0.005	0.64 (0.28, 1.12)	M	21%
Neural	–5.5 (–9.3, − 1.8)	0.006	0.66 (0.27, 1.28)	M	32%
Day 1 (pre-treatment) – Day 3 (follow-up)					
Clean Air	0.8 (–5.6, 4.0)	0.72	–0.07 (–0.63, 0.31)	Nil	17%
Fear	–4.2 (–8.1, − 0.4)	0.03	0.49 (0.11, 0.95)	S	27%
Joy	2.4 (–0.9, 5.8)	0.14	–0.32 (–0.95, 0.14)	S	9%
Neural	0.8 (–4.3, 5.8)	0.75	–0.07 (–0.45, 0.46)	Nil	18%

M diff = Mean difference tested for each odour condition as a within group measure. *P*-value = *p*-value resulting from the paired-sample *t*-test. Mag = Magnitude of change (Nil = negligeable, *d* < 0.2, S = small, 0.2 ≤ *d* < 0.5, M = medium, 0.5 ≤ *d* < 0.8, L = large, *d* ≥ 0.8). RC = Reliable Change index: percentage of subjects reaching clinically meaningful improvement by treatment group^57^.

**Table 3:** Effect size of STAI-Y1 score changes for each odour condition compared to clean air (Day 1, day 2 and follow-up).

Condition	Mean diff. (95% CI)	*P*-value	Cohen’s d (95% CI)	Mag.
Day 1 (pre-post): Clean Air reference change: − 6.25				
Fear	–2.0 (–6.1, 2.2)	0.35	–0.26 (–0.83, 0.31)	S
Joy	0.7 (–3.4, 4.8)	0.73	0.10 (–0.46, 0.66)	Nil
Neutral	–0.4 (–4.5, 3.6)	0.83	–0.06 (–0.62, 0.50)	Nil
Day 2 (pre-post): Clean Air reference change: − 2.54				
Fear	–3.6 (–7.6, 0.2)	0.07	–0.54 (–1.12, 0.03)	M
Joy	–1.0 (–4.9, 3.0)	0.63	–0.17 (–0.73, 0.39)	Nil
Neutral	–3.0 (–7.0, 1.0)	0.14	–0.41 (–1.00, 0.16)	S
Day 1 (pre-treatment) – Day 3 (follow-up): Clean Air reference change: 0.83				
Fear	–5.1 (–10.9, 0.8)	0.09	–0.50 (–1.07, 0.07)	M
Joy	1.6 (–4.2, 7.4)	0.58	0.17 (–0.39, 0.73)	Nil
Neutral	–0.1 (–5.9, 5.8)	0.98	–0.01 (–0.56, 0.55)	Nil

M diff = Mean difference in STAI-Y1 score change for each odour condition compared with clean air, with reference change in the clean air condition reported, calculated with a linear model using odour condition as fixed effect, with confidence intervals derived from the model coefficients. *P*-value = *p*-value resulting from the linear model testing group differences in STAI-Y1 changes. Mag = Magnitude of change (Nil = negligeable, *d* < 0.2, S = small, 0.2 ≤ *d* < 0.5, M = medium, 0.5 ≤ *d* < 0.8, L = large, *d* ≥ 0.8).

### Statistical modelling of state anxiety symptom change

#### Test of primary hypothesis

**Fig. 3:**
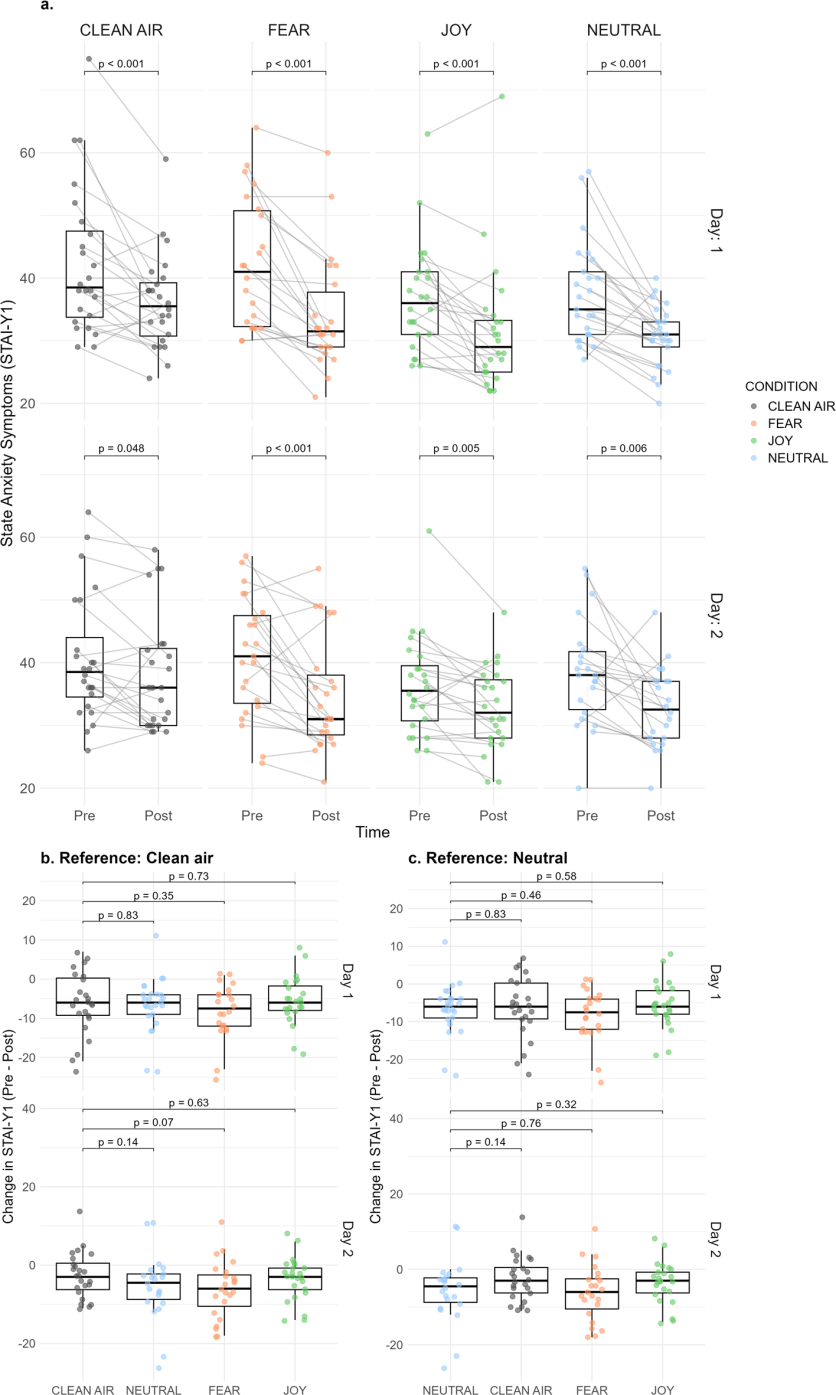
Effects of body odour exposure on state anxiety symptoms. (**a**) Individual changes in STAI-Y1 scores from pre- to post-intervention for each odour condition separately for Day 1 and Day 2. Each point represents one participant, and grey lines connect individual pre–post values. Boxplots display the median and interquartile range; *p*-values reflect results from paired-sample *t*-tests. (**b**) Between-groups comparisons of pre–post STAI-Y1 change scores using clean air as the reference condition. (**c**) Same comparisons using neutral odour as the reference condition.

Linear mixed modelling of the pre-post time points on day 1 and day 2 for all odour groups in a 4 × 2 × 2 model (Odour condition × Time × Day), revealed a main effect of Odour condition (F(3, 93.36) = 2.73, *p* = 0.048) and Time (F(1, 265.6) = 91.91, *p* < 0.001). However, no interaction effect between Odour Condition × Time occurred (*p* = 0.26), rejecting the primary hypothesis. The results are visualised in Fig. 3, whereas Table S2, in the supplementary material, outlines the full analysis output.

### Secondary outcomes

**Fig. 4:**
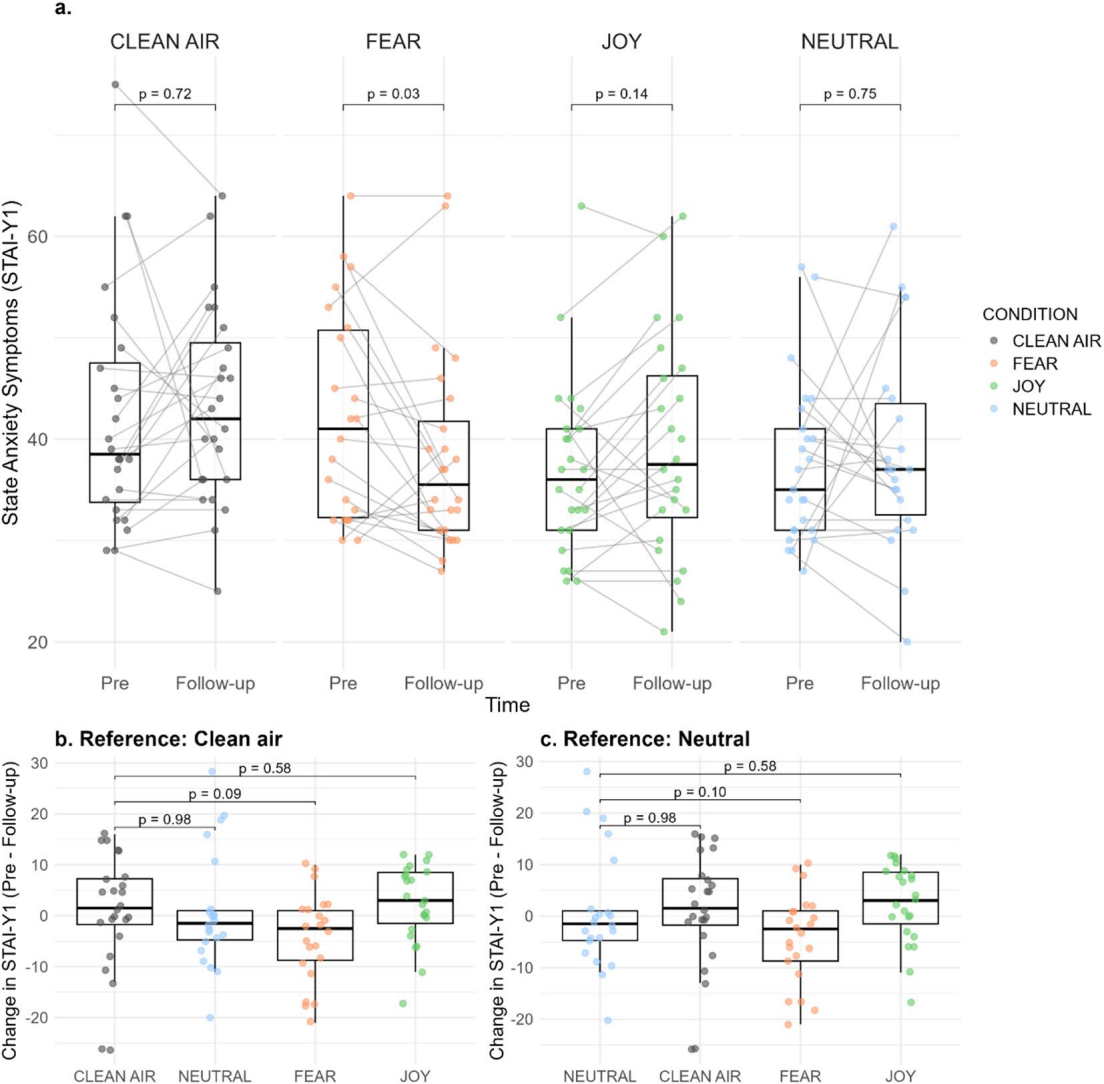
(**a**) Individual changes in STAI-Y1 scores from pre-treatment on day 1 to follow-up on day 3 for each odour condition. Each point represents one participant, and grey lines connect individual values. Boxplots display the median and interquartile range; *p*-values reflect results from paired-sample *t*-tests. (**b**) Between-groups comparisons of pre-treatment and follow-up STAI-Y1 change scores using clean air as the reference condition. (**c**) Between-groups comparisons of pre-treatment and follow-up STAI-Y1 change scores using, using neutral odour as the reference condition.

Following the stress induction on day 2, state anxiety scores increased for all groups, with no differences in change between the odour conditions (*p* = 0.18) (see supplementary material Figure S1). Figure 4 depicts change of state anxiety scores from pre-treatment on day 1 to follow-up (day 3) across all odour groups. Whilst it appeared that the fear odour group had a steeper decline in state anxiety symptoms compared to clean air as well as the other BO conditions (also reflected in the effect size estimates in Tables 2 and 3), linear mixed modelling (4 × 2) did not reveal a significant Odour × Time interaction (*p* = 0.14, see supplementary material Table S3). Moreover, no differences between the groups were seen in changes on the LSAS from pre-treatment to the follow-up assessment (see supplementary material Table S4).

Results regarding perceived usefulness and state mindfulness following the intervention are presented in Table 4. As indicated, a larger proportion of individuals in the odour conditions rated the intervention as more useful (score 7–10) compared with the clean air group. Reflecting this, a significant difference in perceived usefulness emerged between the odour conditions, with holm-adjusted post-hoc analyses indicating that both the fear (*p* = 0.003) and neutral BO groups (*p* = 0.003) rated the intervention as significantly more useful than the clean air group, whereas this difference was not significant for the joy vs. clean air group (*p* = 0.05). Regarding the SMS scale, whilst Kruxal-Wallis examinations of group differences indicated significant differences on SMS-body and marginal differences on SMS-total scales, no Holm-adjusted post-hoc comparisons were significant (SMS Total comparison clean air-fear *p*_*adj*_ = 0.23, clean air-joy *p*_*adj*_ =0.13, clean air-neutral *p*_*adj*_ =0.12; SMS Mind comparison clean air-fear *p*_*adj*_ = 0.17, clean air-joy *p*_*adj*_ =0.23, clean air-neutral *p*_*adj*_ =0.23; SMS Body comparison clean air-fear *p*_*adj*_ = 0.89, clean air-joy *p*_*adj*_ =0.10, clean air-neutral *p*_*adj*_ =0.08).

**Table 4:** Post-treatment state mindfulness scale (SMS) scores and perceived usefulness (PU) scores for each odour condition.

	Clean Air	Fear	Joy	Neutral	*p*
SMS-Mind Mean (SD)	46.9 (9.3)	52.9 (8.5)	52.1 (8.9)	52.5 (10.5)	0.13 ^a^
SMS-Body Mean (SD)	19.6 (4.50)	20.5 (3.80)	22.5(4.8)	22.8 (3.4)	0.02^a^
SMS-Total Mean (SD)	66.5 (12.4)	73.4 (11.2)	74.5 (12.8)	75.2 (12.9)	0.05^a^
PU Mean (SD)	3.5 (2.0)	5.7 (2.1)	5.1 (2.0)	5.7 (2.3)	0.002^a^
PU Score: 0–3	*n* = 16 (66.7%)	*n* = 4 (16.7%)	*n* = 6 (25.0%)	*n* = 5 (22.7%)	
PU Score: 4–6	*n* = 5 (20.8%)	*n* = 10 (41.7%)	*n* = 11 (45.8%)	*n* = 10 (45.5%)	
PU Score: 7–10	*n* = 3 (12.5%)	*n* = 10 (41.7%)	*n* = 7 (29.2%)	*n* = 7 (31.8%)	

^a^ Calculated with Kruskal-Wallis test as data was non-normally distributed. PU score, higher score indicated higher perceived usability.

Table 5 shows Spearman’s correlations between the SMS scale and perceived usefulness (also depicted in the supplementary material, Figure S2). To explore whether the relationship between mindfulness scores and perceived usefulness varied across odour conditions, we conducted three linear models including interaction terms between condition and SMS total (model 1), SMS mind (model 2), and SMS body (model 3) subscale scores. For SMS total and SMS mind, higher mindfulness scores were significantly associated with higher perceived usefulness (SMS total, *p* = 0.01, SMS mind, *p* = 0.02), whereas no effect was seen for SMS body (*p* = 0.07). Whilst condition had a main effect in each model (*p* < 0.05), the interaction terms between SMS scores and condition were not significant (all *p* > 0.6), indicating that the strength of the association between mindfulness and perceived usefulness did not significantly differ across odour conditions.

**Table 5:** Spearman correlations between perceived usefulness and SMS scores (mind, body, and total subscales) across odour conditions.

	SMS Mind		SMS Body		SMS Total	
	Spearman’s ρ	*P*-value	Spearman’s ρ	*P*-value	Spearman’s ρ	*P*-value
Clean Air	0.37	0.07	0.29	0.17	0.43	0.03
Fear	0.70	< 0.001	0.34	0.12	0.65	< 0.001
Joy	0.45	0.03	0.11	0.62	0.36	0.08
Neutral	0.41	0.08	0.47	0.04	0.47	0.04

## Discussion

The aim of the current study was to investigate whether adding human BOs to mindfulness practice could enhance the reductions in state anxiety amongst women meeting criteria for SAD. However, our primary hypothesis that combining mindfulness with BOs, irrespective of their emotional source, would lead to significantly greater reductions in state anxiety symptoms compared to mindfulness alone was not supported. On the contrary, within group analyses indicated significant improvements in state anxiety across all conditions for day 1 and day 2, whereas only the fear BO group showed a significant improvement from day 1 (pre-treatment) to day 3 (follow-up). Likewise, when change in state anxiety scores for each odour condition was compared with change in the clean air condition, no statistically significant differences emerged, for either of the treatment days, nor when change from day 1 (pre-treatment) to day 3 (follow-up) was assessed. However, effect size estimates were noteworthy, particularly when the clean air condition was contrasted to the fear BO group on day 2 and day 1-day 3, with between-groups comparisons reaching a medium effect size. Moreover, whilst more individuals in the fear condition also appeared to have achieved a clinically meaningful reduction in state anxiety symptoms^57^, it should be noted that this number was low across all groups, ranging from 21 to 45% for intervention day 1 and 2, with an even lower number of individuals having maintained this at follow-up on day 3 (ranging from 9 to 27% across the groups).

Regarding study design, methods and procedures, these were shown to be feasible. There was a low number of individuals lost to follow-up during the intervention days (*n* = 2 at day 2), with an additional two individuals not completing the follow-up assessment at day 3, and no participant choosing to actively withdraw from the study. Additionally, no differences were seen between the odour groups (including clean air) regarding perceived intensity, pleasantness, and familiarity of the odours, indicating no marked perceptual differences between the odour conditions that could have influenced our findings. This provides further indications that the odour delivery method was suitable and would be appropriate for future studies.

Based on our previous exploratory study^24^, we speculated that BOs, irrespective of the emotional context in which they have been obtained, are mainly processed as a social stimulus^58,59^, which may strengthen treatment outcome through conveying a social presence. Such interpretations also converge with findings indicating that individuals with SAD report higher positive affect in the presence of others, in comparison to being alone^41^. Contradictive of this, and the emotion contagion hypothesis^7,12,13,18,40^, our current findings provided preliminary indications that fear chemosignals, in conjunction with mindfulness may have potential to enhance its state anxiety reducing effects. However, given that our primary hypothesis was rejected, and the additional benefits of fear BO appearing seemingly weak, these assertions would need to be studied further through a larger well-powered RCT. This would also add important knowledge to a current research gap, given that studies on chemosignal responses for psychiatric patients are rare^5,22^. Also, in contrast to our findings, one of the few studies having evaluated change in anxiety symptoms in response to chemosignals found that healthy females exposed to the BO of anxious males reported increased state anxiety, in contrast to exposure to emotionally neutral BO^18^. Thus, to our knowledge, this and our previous study^24^ were the first to assess changes in self-reported anxiety symptoms among socially anxious females following BO exposure. Moreover, in our studies, we used mindfulness as a contextual element, which may explain the discrepancies with previous findings^18^. Fear chemosignals are also known to strengthen sensory acquisition and attentional resources in receivers, mainly thought to represent an adaptive response to potential danger^60,61^. However, within a mindfulness context, it is plausible that fear chemosignals, through supporting heightened sensory acquisition, could strengthen the ability for focussed attention; one of the key elements within mindfulness practice^62^. Supporting this speculation is research showing that both mindfulness practice^60^ and exposure to fear chemosignals^61^ can strengthen attentional ability by decreasing inattentional blindness. However, it should also be noted that speculations regarding the specificity of the fear BO in relation to neutral and joy BOs should be made with caution, as the somewhat heightened effects seen in the fear condition may be reflective of such BOs being collected from individuals experiencing greater emotional and physiological arousal^63^, potentially having amplified BO production. Moreover, the interpretation that fear BO may have enhanced the ability to practice mindfulness was not supported by our results on the SMS, where no significant differences between the odour conditions were seen on achieved state of mindfulness. On the other hand, our findings regarding perceived usefulness were encouraging, as all BO groups considered the mindfulness practice to be significantly more useful in comparison to the clean air condition. These results provide further support towards continued evaluation of the potential utilisation of chemosignals to enhance mindfulness practice for social anxiety, particularly given the relatively high therapy attrition rates for this patient group^34^.

### Limitations

This study has several limitations that need to be considered. First, whilst we conducted an a-priori power calculation for the current pilot, this was based on a first of its kind, exploratory study^24^. It is therefore probable that this was not a valid representation of the true population value^64^. Conducting the study on a larger sample would likely have yielded more informative conclusions regarding effectiveness. Regarding the collection of BOs used in the current study, whilst it appeared that the mood induction increased the emotion of interest, it should be noted that other emotions were elicited as well (such as fear, as well as anger, disgust, and surprise for the fear condition). Therefore, interpreting each BO condition as representing only the emotion of interest should be done with caution. Moreover, whilst we aimed to induce a happiness emotional state (both to align with previous olfaction research using similar emotion induction procedures^24,40^, and to align with the commonly used term ‘happiness condition’), we acknowledge that happiness as an emotion is complex^65^ and may be difficult to induce through amusing videos. Therefore, we used the term ‘joy’, which more accurately reflects the specific emotional state targeted in the induction.

Additionally, whilst we attempted to avoid potential confounding effects of individual characteristics of BO donors, including sex, by utilising a super-donor design, such effects can nevertheless not be ruled out entirely. Although odour pleasantness was rated on a unidirectional scale (pleasantness only), we did not assess unpleasantness separately; this may limit the sensitivity in detecting subtle aversive reactions, even though the primary aim of the rating was to confirm perceptual equivalence across conditions.

Although no significant differences were found in ratings of familiarity, pleasantness, and intensity for both the body odours and clean air conditions, this does not conclusively imply that the body odour was below the threshold of conscious detection. In the absence of an odorous but non-social control stimulus (e.g. unused cotton pads), we acknowledge that we cannot fully rule out the influence of subtle perceptual cues that may have contributed to the observed effects.

Some critical reflection on the study design is also necessary; The mindfulness intervention was only conducted across two consecutive days, and thus any speculations about benefits of longer-term mindfulness practice together with chemosignals are limited. It is also important to highlight that the current study did not utilise a double-blind randomised controlled trial design. However, whilst a ‘gold-standard’ randomization and blinding method would have been optimal, a quasi-randomised design was chosen to maximise the use of the sweat pads which, we reasoned, was a rational and pragmatic compromise at this pilot stage. Likewise, whilst the sample fulfilled criteria for SAD, the pre-treatment state anxiety scores were in the low-moderate range, which may have led to floor effects. Future studies should therefore consider including individuals with a higher state anxiety symptom profile. Our study was restricted to young women to avoid sex and age related confounds; particularly given that women have been shown to have better olfactory abilities^47^. This further limit the generalisability of our findings and calls on future research to include males.

Finally, it is important to acknowledge that the current study relied on emotion-induced sweat from donors, which would present significant logistical challenges for scaling up future therapies incorporating chemosignals. Likewise, inducing negative emotional states in donors (e.g. to obtain fear chemosignals) to scale up treatments for anxiety raises important ethical concerns. In response to these potential challenges, our research group is conducting work on isolating and synthesising key chemosignal compounds with the goal to create standardised and scalable solutions^66^. However, this work is ongoing, and its potential therapeutic effects are currently being evaluated^67^.

### Conclusion

The current study provides preliminary support for continuing to study the potential of chemosignals to enhance the outcome and perceived usefulness of mindfulness practice for individuals with SAD. Thus, to build on these findings, a larger RCT is warranted, ideally incorporating more mindfulness + BO sessions, to shed further light on the possibility of using human chemosignals for therapeutic purposes, as well as to study mechanisms underlying any potential effects. Such research is particularly paramount, given the increasing rates of anxiety-related disorders across the globe^68^, which highlights the importance of optimising alternative treatment options.

## Supplementary Information

Below is the link to the electronic supplementary material.

Supplementary Material 1

## Data Availability

The datasets analysed during the current study can be made available from the corresponding author on reasonable request (contact: emma.eliasson@ki.se).
