# Supplementary material for: A quasi-randomised pilot study on the efficacy and perceived usefulness of adding chemosignals to mindfulness practice for women with social anxiety
Source: Sci Rep. 2025 Sep 12;15:32459. doi: 10.1038/s41598-025-18246-w (PMC12432134; doi:10.1038/s41598-025-18246-w)

**Supplementary Materials**

Table S1. Emotional ratings before and after watching the videos.

| **Emotional state** | Fear Induction | | | Joy Induction | | | Neutral Induction | | |
| --- | --- | --- | --- | --- | --- | --- | --- | --- | --- |
|  | Mean diff. | ES of change | *p* | Mean diff. | ES of change | *p* | Mean diff. | ES of change | *p* |
| Fear | 42.5 | 1.45 | <.001 | –6.2 | –0.91^a^ | .017 | 1.0 | 0.67^a^ | .423 |
| Anger | 10.7 | 0.76^a^ | .036 | –1.1 | –0.46^a^ | .306 | –1.9 | –0.67^a^ | .423 |
| Disgust | 12.8 | 0.97^a^ | .003 | –0.3 | –0.29^a^ | .590 | –0.4 | –1.00^a^ | 1.00 |
| Surprise | 26.7 | 0.97 | .001 | 10.6 | 0.51^a^ | .125 | 9.4 | 0.33 | .385 |
| Joy | –30.5 | –1.22 | <.001 | 7.9 | 0.69 | .014 | –5.5 | –0.17^a^ | .726 |
| Sadness | 2.3 | 0.33^a^ | .348 | –2.1 | –1.00^a^ | .008 | –0.8 | –0.06 | .865 |
| Amused | –15.4 | –0.54 | .047 | 24.3 | 1.79 | <.001 | –2.5 | –0.13 | .730 |
| Neutral | –36.2 | –1.11 | <.001 | –18.3 | –1.11 | <.001 | 8.1 | 0.46 | .230 |
| Calm | –42.3 | –1.97 | <.001 | –1.1 | –0.18^a^ | .570 | 15.1 | 0.40 | .296 |

Note: Participants’ emotional state (i.e., how much they felt angry, fearful, joyful, sad, disgusted, neutral, surprised, calm, and amused) were assessed using a 100-point Likert scale (from 1, not at all, to 100, very much). ES = Effect Size. ^a^ = Data non-normally distributed, ES calculated using Rank Biserial Correlation. ES of normally distributed data calculated using Cohen’s *d*.

**Sniffin’ Sticks Test description.**

The Sniffin’ Sticks test is a validated and widely used test of olfactory ability (Hummel, Kobal, Gudziol, & Mackay-Sim, 2007). The Sniffin’ Sticks screening procedure for the current study was similar to that described in Cecchetto et al. (2023). The test is composed of three subtests, to assess three different olfactory functions; First, odor identification is measured by presenting 16 common odors, each paired with four verbal and visual descriptors in a multiple forced-choice format (three distractors and one target). Second, odor discrimination is assessed over 16 trials using a three alternative force choice (3AFC) task: for each trial, three pens were presented in random order, two containing the same odor and the third containing the target odor. Third, odor threshold is assessed for n-butanol again with a 3AFC task in which 16 triplets of pens were presented the participants: one containing an odorous solution (target) and two blanks filled with the solvent (distractors). Sixteen dilutions of n-butanol were prepared in a geometric series starting from a 4% n-butanol solution, where participants were asked to identify the odor-containing pen. Reversal of the staircase was triggered when the odor was correctly identified in two successive trials. Threshold was defined as the mean of the last four of seven staircase reversals. In each sub-test, odors were presented in felt-tip pens filled with 4 ml of liquid odorants or odorants dissolved in propylene glycol. For the odor presentation, the cap was removed by the experimenter for approximately 3 seconds and the pen’s tip was placed approximately 2 cm in front of both nostrils. Each sub-test was scored between 1 and 16. A total TDI (Threshold Discrimination Identification) score below 16.5 is within the anosmic range, between 16.5 and 30.5 within the hyposmic range. Only participants with a TDI score of 30.5 or above were included in the study.

Table S2. Linear mixed modelling (4 × 2 × 2) of the pre-post time points across day 1 and day 2 for all odour conditions.

| **Model 1 (N = 98)** | **Sum Sq** | **Mean Sq** | **NumDF** | **DenDF** | **F-value** | ***p*** |
| --- | --- | --- | --- | --- | --- | --- |
| Condition | 258.35 | 86.12 | 3 | 93.36 | 2.73 | 0.05 |
| Time | 2897.91 | 2897.91 | 1 | 265.61 | 91.91 | < .001 |
| Day | 18.11 | 18.11 | 1 | 270.98 | 0.57 | 0.45 |
| Condition:Time | 126.02 | 42.01 | 3 | 265.61 | 1.33 | 0.26 |
| Condition:Day | 117.68 | 39.23 | 3 | 270.87 | 1.24 | 0.29 |
| Time:Day | 117.12 | 117.12 | 1 | 265.61 | 3.71 | 0.06 |
| Condition:Time:Day | 20.52 | 6.84 | 3 | 265.61 | 0.22 | 0.88 |
| *R*^2^ = 0.148 |  |  |  |  |  |  |
| **Model 2 (N = 91)** | **Sum Sq** | **Mean Sq** | **NumDF** | **DenDF** | **F-value** | ***p*** |
| Condition | 247.70 | 82.57 | 3 | 82.21 | 2.63 | 0.06 |
| Time | 2750.04 | 2750.04 | 1 | 254.31 | 87.45 | < .001 |
| Day | 23.91 | 23.91 | 1 | 256.10 | 0.76 | 0.38 |
| Odour intensity | 46.65 | 46.65 | 1 | 81.69 | 1.48 | 0.23 |
| Odour familiarity | 3.01 | 3.01 | 1 | 82.07 | 0.10 | 0.76 |
| Odour pleasantness | 18.48 | 18.48 | 1 | 82.32 | 0.59 | 0.45 |
| Meditation experience | 3.26 | 3.26 | 1 | 82.13 | 0.10 | 0.75 |
| Condition:Time | 121.18 | 40.39 | 3 | 254.31 | 1.28 | 0.28 |
| Condition:Day | 131.64 | 43.88 | 3 | 256.08 | 1.40 | 0.24 |
| Time:Day | 65.06 | 65.06 | 1 | 254.31 | 2.07 | 0.15 |
| Condition:Time:Day | 60.32 | 20.11 | 3 | 254.31 | 0.64 | 0.59 |
| *R*^2^ = 0.162 |  |  |  |  |  |  |
| **Model 3 (N = 65)** | **Sum Sq** | **Mean Sq** | **NumDF** | **DenDF** | **F-value** | ***p*** |
| Condition | 131.12 | 43.71 | 3 | 55.80 | 1.54 | 0.22 |
| Time | 1644.79 | 1644.79 | 1 | 180.78 | 57.77 | < .001 |
| Day | 0.40 | 0.40 | 1 | 181.52 | 0.01 | 0.91 |
| Odour intensity | 4.98 | 4.98 | 1 | 55.57 | 0.17 | 0.68 |
| Odour familiarity | 18.22 | 18.22 | 1 | 55.71 | 0.64 | 0.43 |
| Odour pleasantness | 0.81 | 0.81 | 1 | 55.63 | 0.03 | 0.87 |
| Meditation experience | 10.38 | 10.38 | 1 | 55.62 | 0.36 | 0.55 |
| SOS | 59.35 | 59.35 | 1 | 55.68 | 2.08 | 0.15 |
| Condition:Time | 76.00 | 25.33 | 3 | 180.78 | 0.89 | 0.45 |
| Condition:Day | 217.71 | 72.57 | 3 | 181.43 | 2.55 | 0.06 |
| Time:Day | 22.50 | 22.50 | 1 | 180.78 | 0.79 | 0.38 |
| Condition:Time:Day | 29.03 | 9.68 | 3 | 180.78 | 0.34 | 0.80 |
| *R*^2^ = 0.177 |  |  |  |  |  |  |

Notes: Model 1, full sample with no covariates included. Model 2, Odour ratings and meditation experience included. Model 3 Odour ratings, meditation experience and Social Odour Scale (SOS) included. The inclusion of covariates without (Model 2) and with Model 3) the SOS are presented due to the substantial amount of missing data on the SOS.

Figure S1. Individual changes in STAI-Y1 scores from post-treatment on day 2 to stress-induction on day 2 for each odour condition. Each point represents one participant, and grey lines connect individual values. Boxplots display the median and interquartile range; *p*-values reflect results from paired-sample *t*-tests.


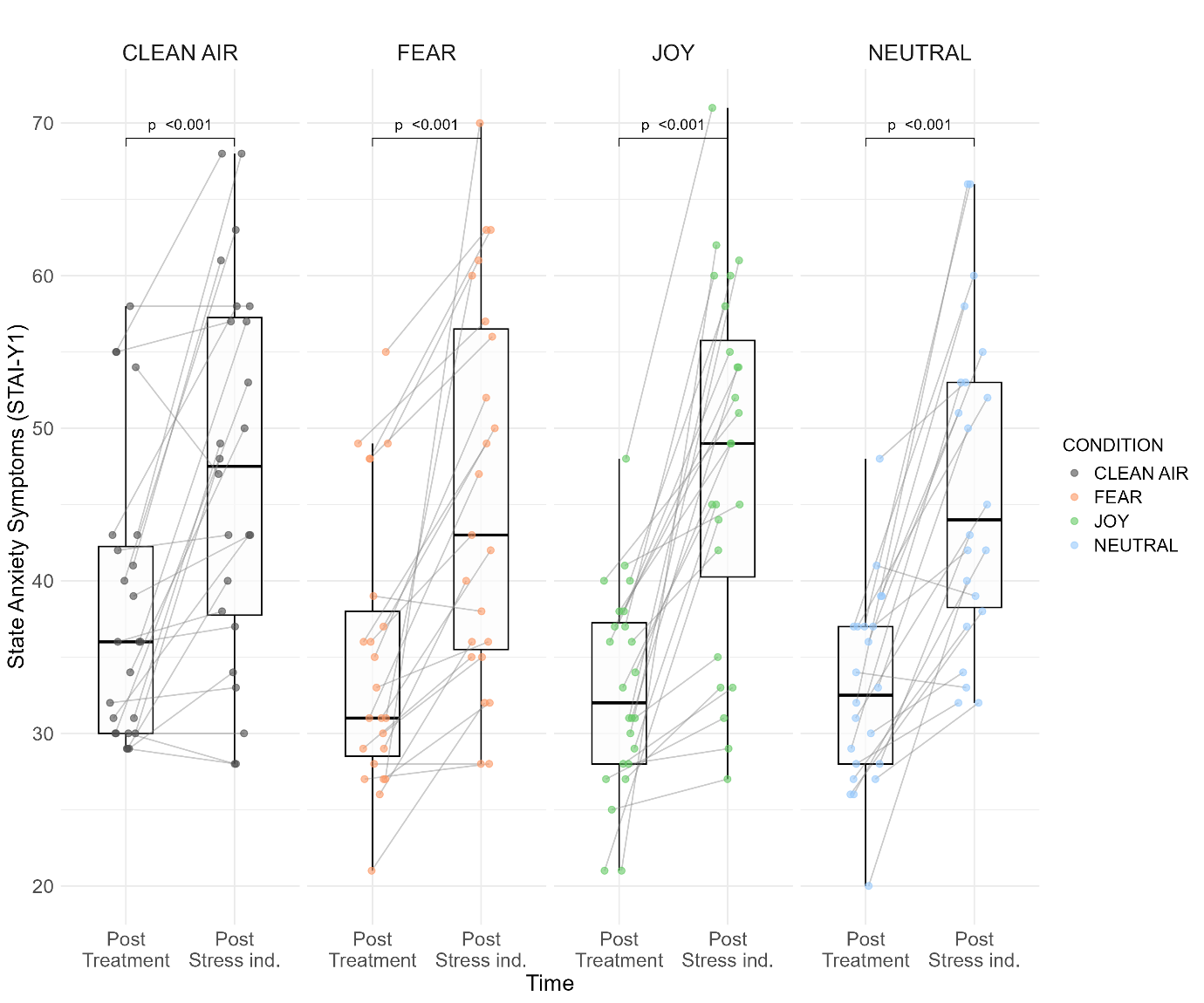


Table S3. Linear mixed modelling of change in STAI scores from baseline to day 3, for all odour conditions (4 × 2 model)

| **Model 1 (N = 98)** | **Sum Sq** | **Mean Sq** | **NumDF** | **DenDF** | **F-value** | ***p*** |
| --- | --- | --- | --- | --- | --- | --- |
| Condition | 252.77 | 84.26 | 3 | 95.24 | 1.72 | 0.17 |
| Time | 0.04 | 0.04 | 1 | 91.56 | 0.001 | 0.98 |
| Condition:Time | 273.59 | 91.20 | 3 | 91.53 | 1.87 | 0.14 |
| *R*^2^ = 0.053 |  |  |  |  |  |  |
| **Model 2 (N = 91)** | **Sum Sq** | **Mean Sq** | **NumDF** | **DenDF** | **F-value** | ***p*** |
| Condition | 230.65 | 76.88 | 3 | 84.08 | 1.59 | 0.20 |
| Time | 8.95 | 8.95 | 1 | 85.34 | 0.19 | 0.67 |
| Odour intensity | 41.39 | 41.39 | 1 | 82.57 | 0.86 | 0.36 |
| Odour familiarity | 64.24 | 64.24 | 1 | 83.07 | 1.33 | 0.25 |
| Odour pleasantness | 48.40 | 48.40 | 1 | 83.74 | 1.00 | 0.32 |
| Med. Experience | 9.15 | 9.15 | 1 | 84.03 | 0.19 | 0.66 |
| Condition:Time | 354.16 | 118.06 | 3 | 85.25 | 2.45 | 0.07 |
| *R*^2^ = 0.086 |  |  |  |  |  |  |
| **Model 3 (N = 65)** | **Sum Sq** | **Mean Sq** | **NumDF** | **DenDF** | **F-value** | ***p*** |
| Condition | 110.87 | 36.96 | 3 | 56.38 | 0.85 | 0.47 |
| Time | 34.59 | 34.59 | 1 | 59.49 | 0.80 | 0.38 |
| Odour intensity | 63.15 | 63.15 | 1 | 55.81 | 1.45 | 0.23 |
| Odour familiarity | 8.96 | 8.96 | 1 | 55.85 | 0.21 | 0.65 |
| Odour pleasantness | 4.52 | 4.52 | 1 | 55.98 | 0.10 | 0.75 |
| Med. Experience | 28.11 | 28.11 | 1 | 56.35 | 0.65 | 0.42 |
| SOS | 42.86 | 42.86 | 1 | 56.05 | 0.99 | 0.33 |
| Condition:Time | 75.94 | 25.31 | 3 | 59.48 | 0.58 | 0.63 |
| *R*^2^ = 0.114 |  |  |  |  |  |  |

Notes: Model 1, full sample with no covariates included. Model 2, Odour ratings and meditation experience included. Model 3 Odour ratings, meditation experience and Social Odour Scale (SOS) included. The inclusion of covariates without (Model 2) and with (Model 3) the SOS are presented due to the substantial amount of missing data for this outcome.

Table S4. Linear Mixed Model output for LSAS scores (model 4 × 2).

| **Model 1 (N = 98)** | **Sum Sq** | **Mean Sq** | **NumDF** | **DenDF** | **F-value** | ***p*** |
| --- | --- | --- | --- | --- | --- | --- |
| Condition | 25.02 | 8.34 | 3 | 93.91 | 0.22 | 0.88 |
| Time | 0.00 | 0.00 | 1 | 86.51 | 0.000 | 1.00 |
| Condition:Time | 156.76 | 52.25 | 3 | 86.50 | 1.40 | 0.25 |
| *R*^2^ = 0.008 |  |  |  |  |  |  |
| **Model 2 (N = 91)** | **Sum Sq** | **Mean Sq** | **NumDF** | **DenDF** | **F-value** | ***p*** |
| Condition | 44.01 | 14.67 | 3 | 83.17 | 0.42 | 0.74 |
| Time | 12.04 | 12.03 | 1 | 82.51 | 0.35 | 0.56 |
| Odour intensity | 2.84 | 2.84 | 1 | 82.56 | 0.08 | 0.78 |
| Odour familiarity | 164.90 | 164.90 | 1 | 82.96 | 4.76 | 0.03 |
| Odour pleasantness | 159.68 | 159.68 | 1 | 83.09 | 4.61 | 0.03 |
| Med. Experience | 13.25 | 13.25 | 1 | 83.15 | 0.38 | 0.54 |
| Condition:Time | 209.75 | 69.92 | 3 | 82.49 | 2.02 | 0.12 |
| *R*^2^ = 0.077 |  |  |  |  |  |  |
| **Model 3 (N = 65)** | **Sum Sq** | **Mean Sq** | **NumDF** | **DenDF** | **F-value** | ***p*** |
| Condition | 65.76 | 21.92 | 3 | 56.03 | 0.65 | 0.59 |
| Time | 18.77 | 18.77 | 1 | 58.24 | 0.56 | 0.46 |
| Odour intensity | 1.17 | 1.17 | 1 | 55.90 | 0.03 | 0.85 |
| Odour familiarity | 196.74 | 196.74 | 1 | 55.91 | 5.83 | 0.02 |
| Odour pleasantness | 199.83 | 199.83 | 1 | 55.94 | 5.92 | 0.02 |
| Med. Experience | 19.66 | 19.66 | 1 | 56.02 | 0.58 | 0.45 |
| SOS | 0.94 | 0.94 | 1 | 55.95 | 0.03 | 0.87 |
| Condition:Time | 139.82 | 46.61 | 3 | 58.24 | 1.38 | 0.26 |
| *R*^2^ = 0.150 |  |  |  |  |  |  |

Notes: Model 1, full sample with no covariates included. Model 2, Odour ratings and meditation experience included. Model 3 Odour ratings, meditation experience and Social Odour Scale (SOS) included. The inclusion of covariates without (Model 2) and with (Model 3) the SOS are presented due to the substantial amount of missing data for this outcome.

Figure S2. Association between perceived usefulness and SMS total scores, as well as SMS body and mind subscales, across odour conditions. Linear regression lines are shown for each condition, with corresponding *p*-values derived from Spearman’s rank correlation test (non-normally distributed data).


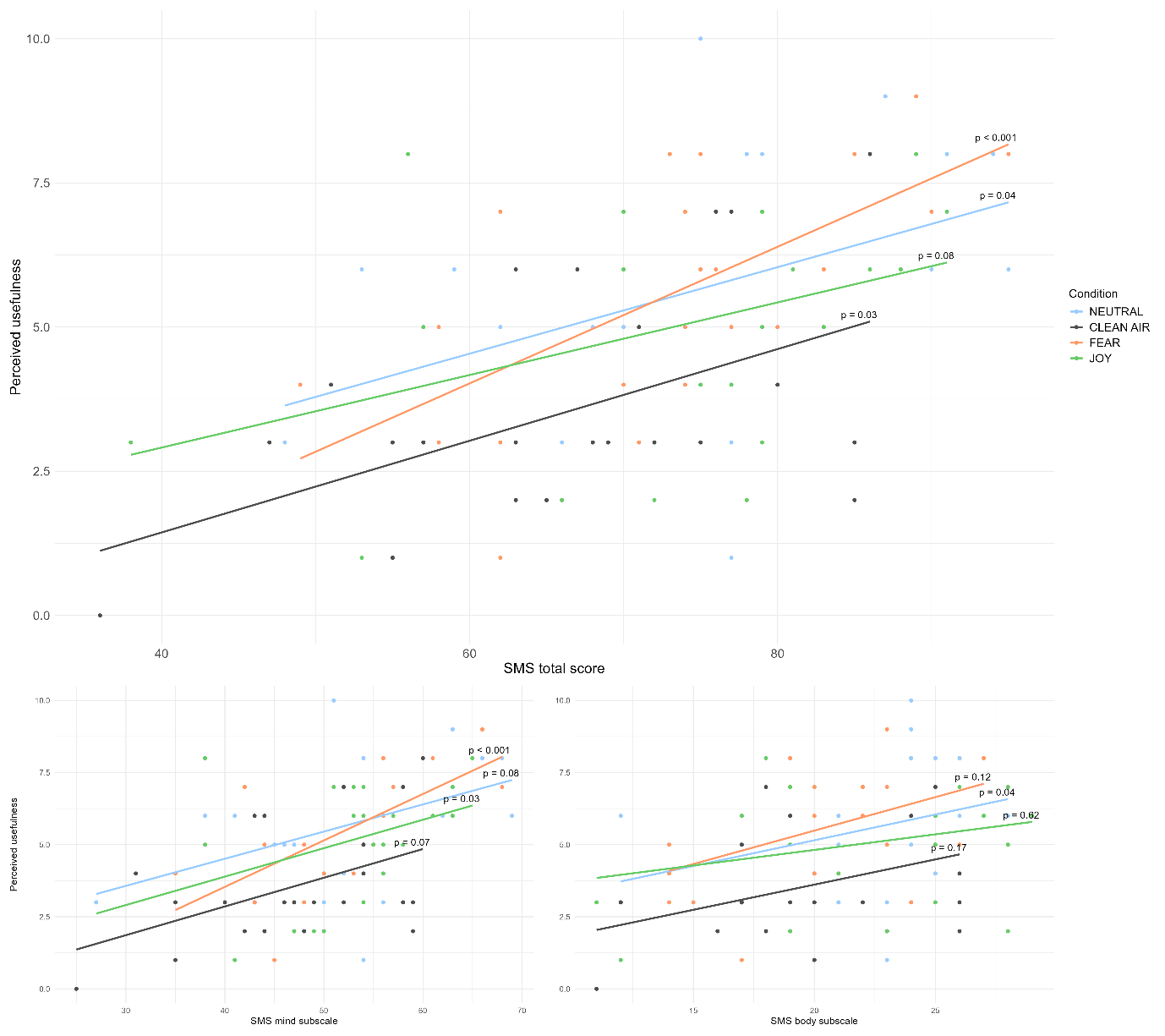

Supplement: Supplementary file 1 — Supplementary Material 1 [file 41598_2025_18246_MOESM1_ESM.docx]
